# Cerebral Venous Sinus Thrombosis Related to Iron-Deficiency Anemia

**DOI:** 10.7759/cureus.8917

**Published:** 2020-06-29

**Authors:** Prachi Shah, Dan Nguyen, Brian Berman

**Affiliations:** 1 Pediatrics, Oakland University William Beaumont School of Medicine, Royal Oak, USA; 2 Emergency Medicine, Oakland University William Beaumont School of Medicine, Royal Oak, USA; 3 Emergency Medicine, William Beaumont Hospital, Royal Oak, USA

**Keywords:** cerebral venous sinus thrombosis, iron-deficiency anemia

## Abstract

Cerebral venous sinus thrombosis (CVST) is an uncommon diagnosis associated with life-threatening and long-term neurological consequences in children. It is characterized by non-specific symptoms, including fever, altered mental status and focal neurological deficits. Etiologic factors include infection, trauma, prothrombotic disorders and importantly, iron-deficiency anemia. Iron-deficiency anemia is a preventable risk factor of CVST as it is commonly caused by excessive cow milk consumption in infants and toddlers. Diagnosis is mainly made with MRI and magnetic resonance venography (MRV). Prompt anticoagulation is a key therapeutic intervention, and in the case of associated iron deficiency, iron repletion and elimination or limitation of milk from diet are also required. Thus, it is essential for physicians to have a high level of suspicion to diagnose CVST, given its non-specific presentation. This is a case report of a two-year-old boy who presented to the pediatric emergency department with vomiting and altered mental status and was ultimately diagnosed with CVST.

## Introduction

Cerebral venous sinus thrombosis (CVST) is an uncommon disorder that can be linked to adverse neurological outcomes. It is characterized by the obstruction of blood flow in cerebral veins or major sinuses in the brain [1]. The occurrence of CVST incidence is 0.67 cases for 100,000 children per year, with neonates more commonly affected [2]. Presenting symptoms can be non-specific and include headache, lethargy, vomiting, altered mental status, seizures and focal neurological deficits [3]. CVST should be included in the differential diagnosis of pediatric patients presenting acutely with seizures, stroke, headache, non-traumatic coma and pseudotumor cerebri [4]. Moreover, CVST has multiple etiologies, including infections, trauma, recent intracranial surgery, prothrombotic disorders and iron-deficiency anemia. Since symptoms can be subtle and variable, a high index of suspicion is required to diagnose CVST early. We present a case of a two-year-old boy with vomiting and altered mental status, who was diagnosed with CVST in the setting of iron-deficiency anemia.

## Case presentation

A previously healthy two-year-old male presented to the pediatric emergency department (ED) with nausea, vomiting and diarrhea. He was in his usual state of good health, until one day prior to presentation, when he had an episode of non-bloody diarrhea and non-bloody, non-bilious emesis. On the day of presentation, he had three additional episodes of non-bloody, non-bilious vomiting. He appeared fatigued with decreased energy levels. Parents denied any history of fever, abdominal pain, hematuria, trauma or drug exposure. His diet was primarily comprised of cow’s milk, with little solid food intake. On evaluation, he had a rectal temperature of 96.8°F, respiratory rate of 18 breaths per minute and oxygen saturation of 99% on room air. Mild bradycardia was noted with a heart rate of 82 beats per minute. His head was normocephalic and atraumatic. His mucous membranes appeared dry, and his skin was pale and warm. He appeared lethargic and listless with minimal arousal to stimulus. His Glasgow Coma Scale (GCS) score was 13, with the eye, verbal and motor subscores of 3, 5 and 6, respectively. He exhibited normal muscle tone with intact reflexes. Cranial nerve and sensory exam were grossly normal.

**Figure 1:**
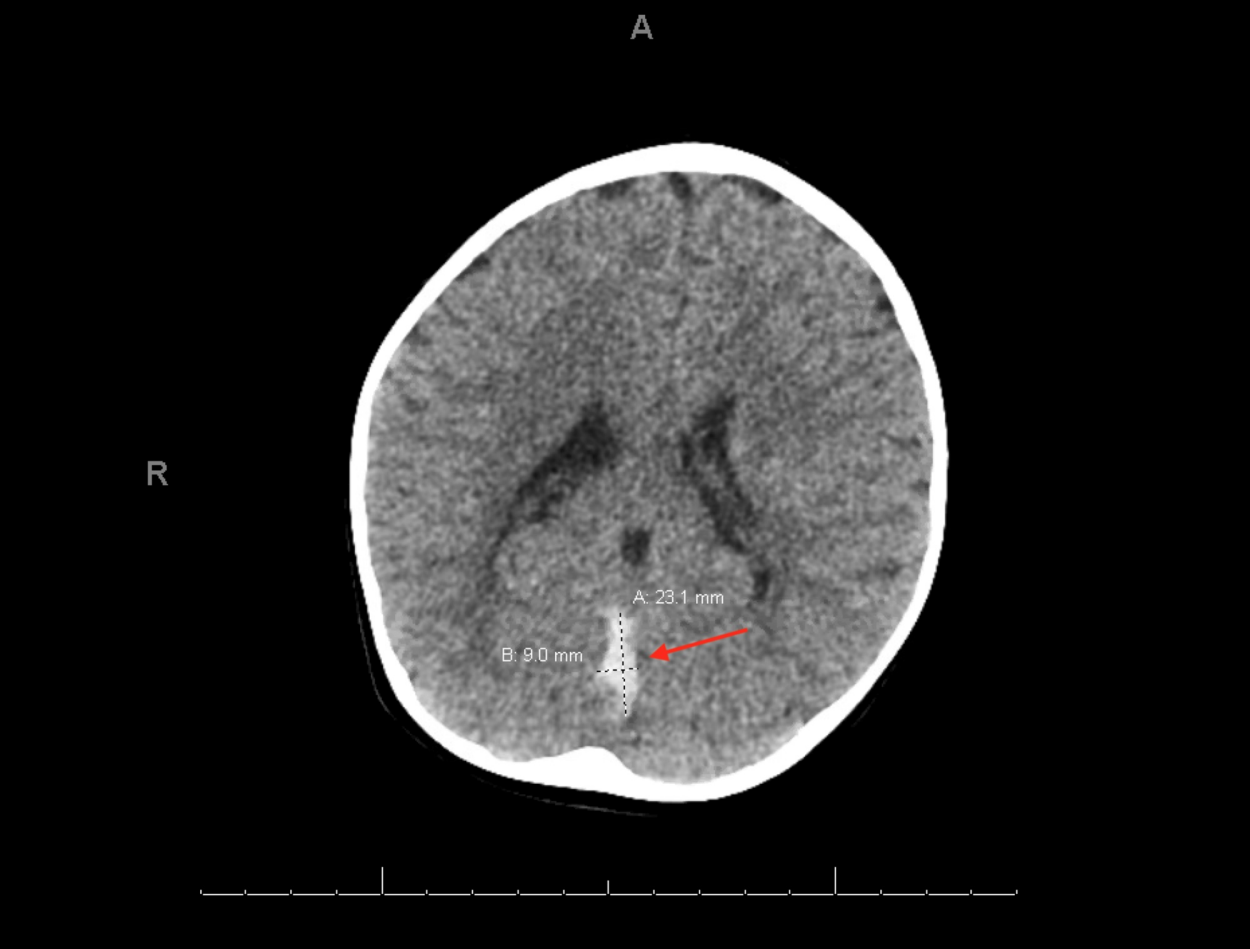
Brain CT scan showing hyperdense collection within the posterior interhemispheric region.

Given his presentation of lethargy and multiple episodes of emesis, he received a bolus of normal saline and ondansetron. Basic metabolic panel was within normal limits. Urinalysis was consistent with dehydration and showed elevated ketones, with no red blood cells (RBCs), white blood cells (WBCs) or nitrites. A complete blood count demonstrated moderate microcytic anemia with a hemoglobin of 8.1 g/dL, mean corpuscular volume 54 fL, mean corpuscular hemoglobin concentration of 27 g/dL, WBC count 13.2 × 10^9^/L with an absolute neutrophil count of 10.3 × 10^9^/L and platelet count of 515,000 × 10^9^/L. Reticulocyte hemoglobin concentration was markedly reduced at 13.7 pg (28.5-37.9 pg) with normal haptoglobin of 95 mg/dL (40-250 mg/dL) and normal lactate dehydrogenase of 248 U/L (155-345 U/L). The serum iron level was reduced at 11 µg/dL (65-175 µg/dL) with increased total iron-binding capacity of 496 µg/dL (250-425 µg/dL), and decreased iron saturation of 2% (15%-50%). His ferritin was decreased at 4.2 ng/mL (14-338 ng/dL). Peripheral blood smear showed hypochromia with ovalocytes, tear drop cells and occasional reactive lymphocytes. Abdominal X-ray and ultrasound were unremarkable. Given his mental status, CT of the brain was obtained and revealed a 2.3 cm by 0.9 cm hyperdense collection within the posterior interhemispheric region (Figure 1). He was admitted to the pediatric intensive care unit (PICU) for further evaluation and management.

**Figure 2:**
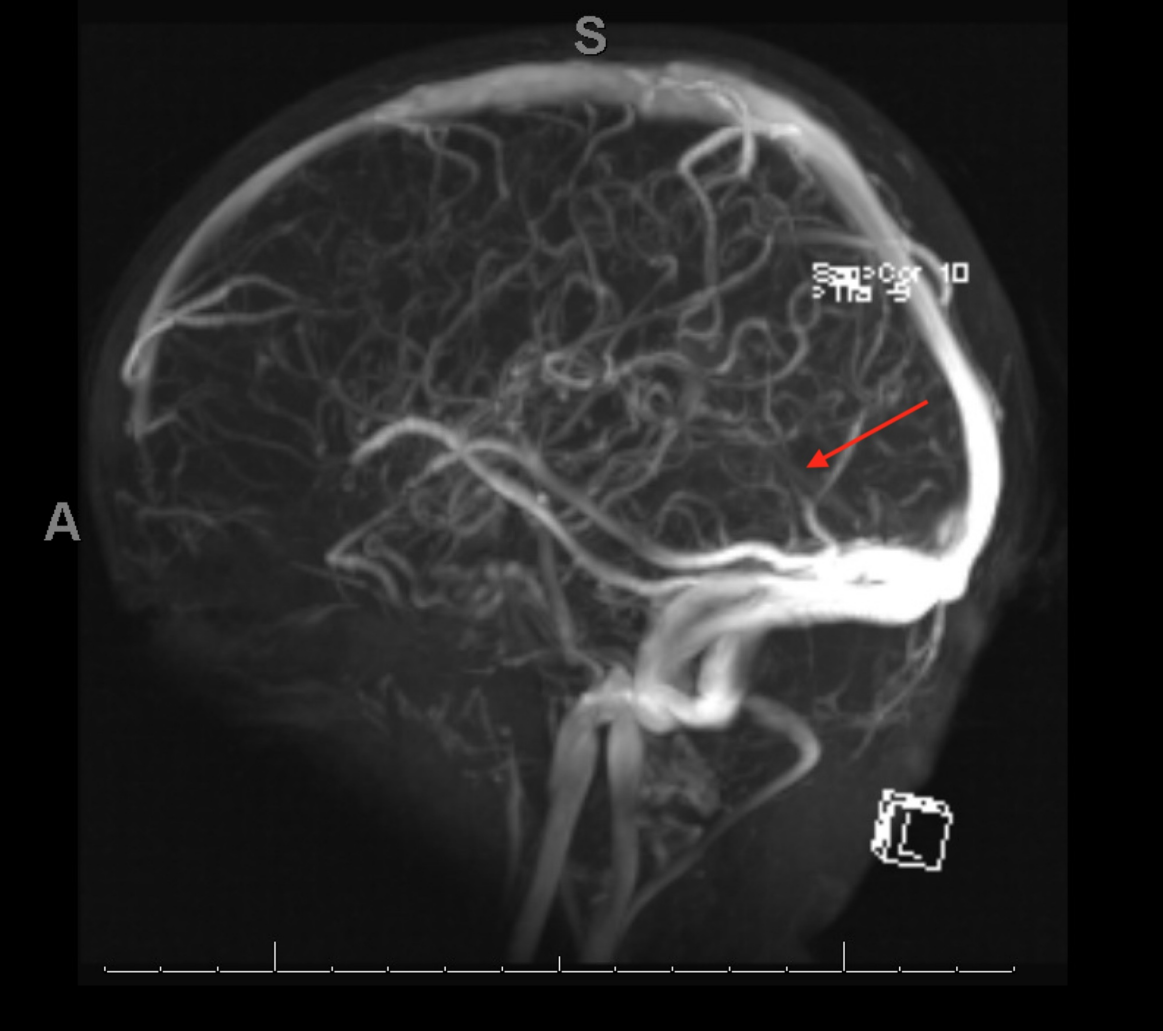
Brain magnetic resonance venography showing thrombosis of the straight sinus with decrease of flow.

In the PICU, MRI and magnetic resonance venography (MRV) of the brain were ordered due to the concern for possible thrombosis or acute subdural hemorrhage. Brain MRV demonstrated thrombosis of the straight sinus, with significantly reduced flow (Figure 2). The patient was subsequently evaluated by the pediatric hematology service and was started on subcutaneous enoxaparin 1 mg/kg/dose twice daily. He was also started on oral iron supplementation of 30 mg twice daily, and parents were advised to eliminate milk from the child’s diet. Although the patient initially had mild leukocytosis, antibiotics were deferred as he remained afebrile and did not have any evidence of sepsis or overt infections like urinary tract infection, meningitis or pneumonia. Moreover, to determine the cause of the venous sinus thrombosis, additional studies, including lupus anticoagulant, antiphospholipid antibody, homocysteine, lipoprotein A, factor V Leiden mutation and prothrombin genotype, were obtained. The day following presentation, his clinical status was significantly improved, with no vomiting and normal sensorium. The child was discharged three days later with the plan to continue anticoagulation with enoxaparin and follow-up in hematology clinic.

**Figure 3:**
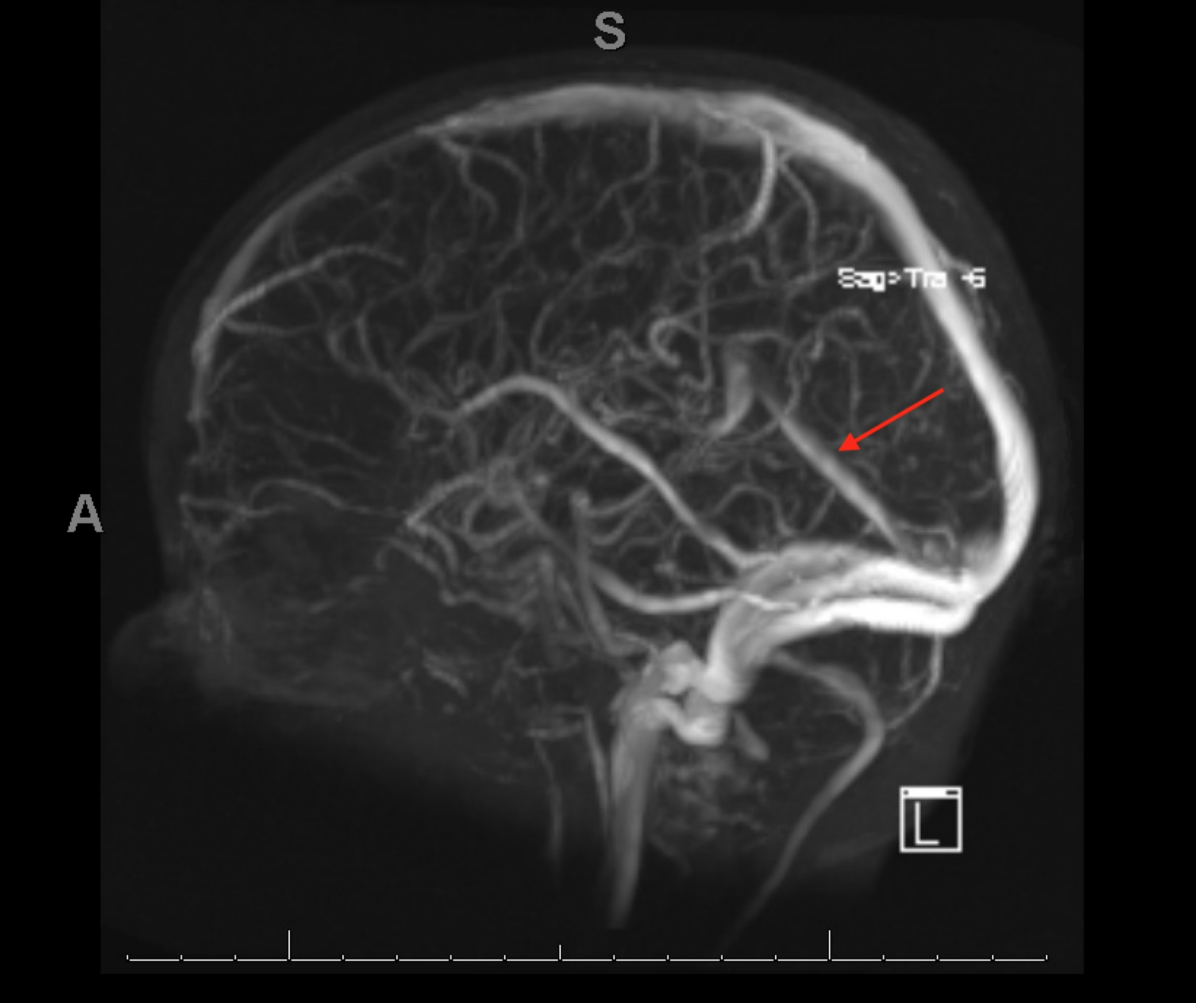
Brain magnetic resonance venography showing resolution of thrombosed straight sinus after three months of enoxaparin therapy, iron supplementation and elimination of milk from diet.

When seen four days following discharge, he was found to have significant improvement with full neurological recovery. Enoxaparin was continued. The child tolerated oral iron poorly and was given several doses of intravenous iron sucrose with an excellent response. Hematologic studies returned to normal over the course of several weeks. He remained on a milk-free diet. Three months following presentation, a repeat MRI and MRV brain demonstrated complete resolution of the venous sinus thrombosis; enoxaparin was discontinued (Figure 3). The hyperthrombotic evaluation included factor V Leiden and prothrombin gene mutation, homocysteine, lipoprotein A, lupus anticoagulant, protein S, C anti-thrombin III, and factor VIII level, all of which were all normal. This suggests that CVST was likely precipitated by iron-deficiency anemia in this patient. He has remained well without recurrence of anemia or neurological symptoms. 

## Discussion

CVST is an uncommon disorder that can be linked to adverse neurological outcomes. It is characterized by the obstruction of blood flow in cerebral veins or major sinuses in the brain [1]. Clinical presentation can be non-specific with patients experiencing symptoms like headaches, vomiting, lethargy, altered mental status, seizures and focal neurological deficits [3]. Although symptoms are not age specific, neonates are more likely to present with dehydration and seizures [2]. Older children are more likely to present with vomiting and altered mental status as seen in our patient [3]. Since symptoms can be subtle and variable, a high index of suspicion is required to diagnose CVST early. Furthermore, CVST often has a multifactorial etiology in children. It can be precipitated by common head and neck infections, head trauma, recent intracranial surgery and autoimmune disorders. Congenital cardiac disease, nephrotic syndrome and malignancies, including leukemia and lymphoma, can also predispose patients to CVST [3]. Prothrombotic disorders increase the overall risk of thrombosis and must also be considered. Factor V Leiden mutation, prothrombin gene mutation, lupus anticoagulant, lipoprotein A, and protein C and S deficiencies have been linked to CVST [2,5].

CVST has been associated with anemia [3]. Most particularly, iron-deficiency anemia can contribute to a hypercoagulable state and predispose children to develop CVST [6]. Anemia leads to decreased oxygen-carrying capacity and increased cerebral blood flow. Iron-deficient red cells have decreased deformability. This results in turbulent flow, activating the coagulation cascade and promoting thrombi formation [7]. Additionally, iron participates in the regulation of platelet levels and helps maintain steady state by inhibiting production. The consequent thrombocytosis promotes thrombosis [7]. Our patient was clearly iron deficient with an elevated platelet count. Thus, it is essential to obtain a complete blood count and iron studies to determine the presence of iron-deficiency anemia when considering a diagnosis of CVST.

Few studies have identified iron-deficiency anemia as a cause of CVST. In these cases, patients presented with varying symptoms, including emesis, diarrhea, lethargy, fever and seizures [7,8]. Similar to our case, they had microcytic anemia and thrombocytosis. Most cases also had an unremarkable evaluation for prothrombotic disorders, suggesting that iron-deficiency anemia was a key etiologic factor [8]. Interestingly, these reports described large cow milk consumption as a cause of the iron-deficiency anemia, as observed in our patient [7,8]. An infant/toddler diet comprised largely of cow milk leads to nutritional deficiency of iron mainly due to the inherent low iron content of cow milk, and requires supplementation to meet the body’s iron requirement. Additionally, cow milk can result in occult stool blood loss due to milk protein-induced, clinically silent enterocolitis. Cow milk’s high calcium and casein content also inhibit the absorption of iron and further worsen the anemia [9].

CVST is primarily diagnosed using imaging modalities, including CT, MRI and MRV. MRI and MRV are preferred in children because CT has lower sensitivity and higher rate of false positives [2]. No clinical trials studying the treatment of pediatric CVST or its association with iron-deficiency anemia are available, and the current therapeutic approach is based on adult clinical trials [2]. Initial treatment includes stabilization and symptomatic care, including rehydration, and antibiotics and anticonvulsants if indicated. Importantly, patients are started on anticoagulants, primarily unfractionated heparin or low molecular weight heparin (LMWH). Anticoagulation with LMWH or warfarin is continued for a period of not less than three to six months, with routine monitoring of anti-factor Xa and international normalized ratio, respectively. Despite the inherent risk of hemorrhage, anticoagulation therapy is generally deemed safe in children, and untreated CVST increases the risk of propagation of the thrombus with resultant brain injury [10].

Patients require repeat MRI and MRV to monitor the degree of resolution of thrombosis, which will help determine the appropriate length of anticoagulation therapy [3]. Close monitoring is required as mortality for CVST ranges between 8% and 12% [1]. Neurological deficits, including cognitive impairment, developmental delays and sensorimotor deficits, can persist long term in some cases [3]. Poorer outcomes are associated with younger age, and those presenting with seizures, infarcts or altered level of consciousness [1]. Patients treated with anticoagulation have better cognitive outcomes and lower risk of recurrence [3].

In summary, CVST is an uncommon diagnosis associated with potentially life-threatening and long-term neurological consequences in children that warrants a high index of suspicion for diagnosis, given its non-specific presentation. Iron-deficiency anemia caused by excessive cow milk consumption serves as an important preventable risk factor. Although previous case reports have identified this relationship, awareness among physicians caring for children is limited.

## Conclusions

CVST is an uncommon disorder characterized by non-specific symptoms including fever, vomiting, altered mental status and focal neurological deficits. Etiologic factors include regional or systemic infection, trauma, prothrombotic disorders and importantly, iron-deficiency anemia. Increased consumption of cow milk is an important cause of iron-deficiency anemia in infants/toddlers. A high level of suspicion is required for diagnosis of CVST, which is established with MRI and MRV. Prompt anticoagulation is a key therapeutic intervention, and in the case of associated iron deficiency, iron repletion and elimination or limitation of milk from diet are also required.
